# Reasonable access: important characteristics and perceived quality of legal and illegal sources of cannabis for medical purposes in Canada

**DOI:** 10.1186/s42238-023-00185-w

**Published:** 2023-06-09

**Authors:** N. Rielle Capler, Lynda G. Balneaves, Jane A. Buxton, Thomas Kerr

**Affiliations:** 1 School of Population and Public Health, Faculty of Medicine, University of British Columbia, 2206 East Mall, Musqueam Traditional Territory, Vancouver, BC V6T 1Z3 Canada; 2 College of Nursing, University of Manitoba, Room 495, 89 Curry Place, Winnipeg, MB R3T 2N2 Canada; 3 British Columbia Centre for Disease Control, 655 West 12th Avenue, Vancouver, BC V5Z 4R4 Canada; 4 British Columbia Centre on Substance Use, 400-1045 Howe Street, Vancouver, BC V6Z 2A9 Canada; 5 Department of Medicine, University of British Columbia, St. Paul’s Hospital, 608-1081 Burrard Street, Vancouver, BC V6Z 1Y6 Canada

**Keywords:** Medical cannabis, Cannabis, Access to healthcare, Legal and illegal cannabis sources, Cannabis products and services, Reasonable access

## Abstract

**Background:**

Throughout the past two decades of legal medical cannabis in Canada, individuals have experienced challenges related to accessing legal sources of cannabis for medical purposes. The objective of our study was to examine the sources of cannabis accessed by individuals authorized to use medical cannabis and to identify possible reasons for their use of illegal sources.

**Methods:**

Individuals who participated in the Cannabis Access Regulations Study (CANARY), a national cross-sectional survey launched in 2014, and indicated they were currently authorized to use cannabis for medical purposes in Canada were included in this study. We assessed differences between participants accessing cannabis from only legal sources versus from illegal sources in relation to sociodemographic characteristics, health-related factors, and characteristics of medical cannabis they considered important. A secondary analysis assessed differences in satisfaction with various dimensions of cannabis products and services provided by legal versus illegal sources.

**Results:**

Half of the 237 study participants accessed cannabis from illegal sources. Individuals accessing cannabis from illegal sources were significantly more likely to value pesticide-free products, access to a variety of strains, ability to select strain and dosage, ability to observe and smell cannabis, availability in a dispensary, and availability in small quantities than did individuals accessing cannabis from only legal sources (all *p* < 0.05). Additionally, participants gave significantly higher satisfaction scores to illegal sources than to legal sources on service-related dimensions of cannabis access (all *p* < 0.05).

**Conclusion:**

Our findings contribute to an understanding of reasonable access to medical cannabis from a patient perspective and how to assess whether it has been achieved. Characteristics of cannabis products and services valued by patients and appropriate to their needs should be incorporated into legal medical cannabis programs to promote the use of legal medical sources. While pertaining specifically to medical use of cannabis in Canada, the findings of this study may also be instructive for understanding the use of illegal cannabis sources for non-medical purposes in Canada and provide insight for other jurisdictions implementing cannabis regulations for both medical and non-medical purposes.

**Supplementary Information:**

The online version contains supplementary material available at 10.1186/s42238-023-00185-w.

## Introduction

On a global level, there is growing recognition of the medicinal value of cannabis (United Nations Commission on Narcotic Drugs, 2020). In response, an increasing number of countries have enacted medical cannabis laws as a means of ensuring access (Aguilar, Gutiérrez, Sánchez, & Nougier, 2018; Schlag 2020). This is true of Canada, where it is estimated that over 1.5 million people, or 5.5% of the population over the age of 15, currently use cannabis for medical purposes (Canadian Centre on Substance Abuse, 2020; Government of Canada 2019b). Canada was one of the first countries to introduce regulations to govern the possession, production, and distribution of medical cannabis. Providing reasonable access to health care is a primary objective of Canada’s health care policy (Government of Canada, 1985). Beginning in 2001, in response to a court ruling establishing a constitution right to access cannabis for medical purposes, the Canadian government’s department of health, Health Canada, initiated a succession of regulations intended to provide reasonable access to cannabis for those in medical need. Each set of regulations stipulated new legal sources in an evolving landscape of medical cannabis; however, reasonable access to medical cannabis has not been defined nor measured.

The first set of Canadian medical cannabis regulations, the *Marihuana Medical Access Regulations* (MMAR), were implemented in 2001 (Government of Canada, 2001). Under the MMAR, individuals who were authorized by Health Canada to use medical cannabis had the option to produce their own supply of cannabis with a personal production license, designate someone to grow on their behalf with a designated production license, or acquire cannabis through mail-order from Health Canada’s sole contracted supplier at the time. The second set of regulations, the *Marihuana for Medical Purposes Regulations* (MMPR), were enacted in 2013, introducing federally licensed producers (LPs) as the only legal source of medical cannabis (Government of Canada 2013), replacing personal and designated production. However, a court injunction in 2014 (*Allard et al. v. Canada*, 2014) delayed the repeal of the MMAR until a court case questioning the constitutionality of removing production licenses under the MMPR could be heard. This complex policy transition from the MMAR to the MMPR resulted in cannabis sources associated with both regulations (i.e., personal production, designated production, Health Canada’s contracted supplier, and LPs) being available to individuals authorized under the respective regulations from 2014 to 2016. Following a court decision in 2016 supporting personal and designated production (*Allard et al. v. Canada*, 2016), a third set of regulations, the *Access to Cannabis for Medical Purposes Regulations* (ACMPR), replaced both the MMAR and MMPR, reinstating personal and designated production options alongside an expanding number of LPs (Government of Canada 2016).

In the current context, a slightly modified version of the ACMPR with the same legal sources of medical cannabis was subsumed under the Cannabis Act and Regulations enacted in 2018 (Government of Canada 2018b, 2018c). This new legislation (referred hereafter as “cannabis legalization”) legalized cannabis for non-medical (i.e., recreational) purposes in Canada and provided new legal sources of non-medical cannabis to adults, including retail storefronts, online sales, and personal cultivation. The federal government committed to a review of the medical cannabis program 5 years after legalization (Government of Canada Standing Committee on Health 2017). A legislative review of the Cannabis Act, including a review of legalization and regulation of cannabis for medical purposes, was announced in September 2022 (Government of Canada 2022a). The legislative review will culminate in a report to Parliament no later than 18 months after the start of the review and may lead to regulatory or programmatic changes (Government of Canada 2023). See Table 1 for the timeline of regulations and related legal sources of cannabis.

**Table 1:** Timeline of regulations and legal sources of cannabis

**Regulations**	**Years**	**Legal sources of cannabis**
MMAR	2001–2016	▪ Personal production with license ▪ Designated producer ▪ Health Canada supplier (starting 2003)
MMPR	2014–2016	▪ Licensed producers
ACMPR	2016–2018	▪ Personal production with license ▪ Designated producer ▪ Licensed producers
Cannabis Act	2018 to present	▪ Personal production with license (medical) ▪ Designated producer (medical) ▪ Licensed producers (medical) ▪ Online and storefront retailers (non-medical) ▪ Personal cultivation (non-medical)

Despite the past two decades of legal medical cannabis in Canada, across four regulatory programs providing various legal sources of cannabis, individuals using cannabis for medical purposes have continued to procure cannabis from illegal sources (i.e., cannabis dispensaries, production without a license, close friend/family, acquaintance/dealer, and unfamiliar street source). In part, this stems from the fact that access to legal sources is restricted to individuals who are authorized under the federal regulations, and most Canadians who use cannabis for medical purposes do so without federal approval. At the time of the MMAR, only 5% of individuals who reported medical cannabis use were authorized to possess cannabis (Belle-Isle et al. 2014). Under the current Cannabis Act, a similar proportion of individuals who report using medical cannabis (6%) are registered with an LP, although 22% report holding a document from a healthcare professional (Government of Canada 2022c). However, even individuals who have obtained the necessary authorization for medical cannabis have been found to utilize illegal sources. Only 20% of authorized users under the MMAR were accessing cannabis exclusively from legal sources (Belle-Isle et al. 2014) and a 2021 national survey revealed that Canadians continue to report purchasing cannabis for medical use from illegal sources (Government of Canada 2021a). This phenomenon is not unique to Canada; participants in a Californian study who had access to legal sources of medical cannabis reported supplementing with purchases from illegal sources (Reed et al. 2020).

While previous studies of medical cannabis access in Canada have focused mainly on barriers to accessing the federal medical cannabis program (Belle-Isle et al. 2014), to our knowledge, no studies have investigated if reasonable access has been achieved in Canada. This study sought to understand reasonable access to medical cannabis from a patient-centered perspective and to fill the gap in research on medical cannabis access after the introduction of LPs. To achieve these aims, the objective of this study was to identify patient and health service-related factors associated with accessing cannabis for medical purposes from legal and illegal sources among adults authorized to use medical cannabis in Canada.

## Methods

### Study design and conceptual framework

This study was part of the Cannabis Access Regulations Study (CANARY), a national cross-sectional study launched in 2014 to examine access to medical cannabis in Canada 6–9 months post-introduction of the MMPR. Eligibility criteria for the CANARY study included self-reporting a diagnosis of a medical condition, living in Canada, being at least 19 years of age, and being able to speak and read English or French. A variety of recruitment strategies were used, including letters of invitation sent via email by national patient advocacy groups, advertisements in medical cannabis advocacy group newsletters, websites and other social media, and letters of invitation distributed through LPs, compassion clubs, and community-based dispensaries (i.e., illegal storefronts, hereafter both referred to as dispensaries) from across Canada. Concerted effort was made to obtain representativeness in the convenience sample with respect to geographical location and language. As part of the CANARY study, a national sample of adults completed an online survey (*N*=369). Ethics approval for the CANARY study was obtained from the Behavioural Review Ethics Board at the University of British Columbia in Vancouver, Canada (certificate # H13-03370).

**Fig. 1:**
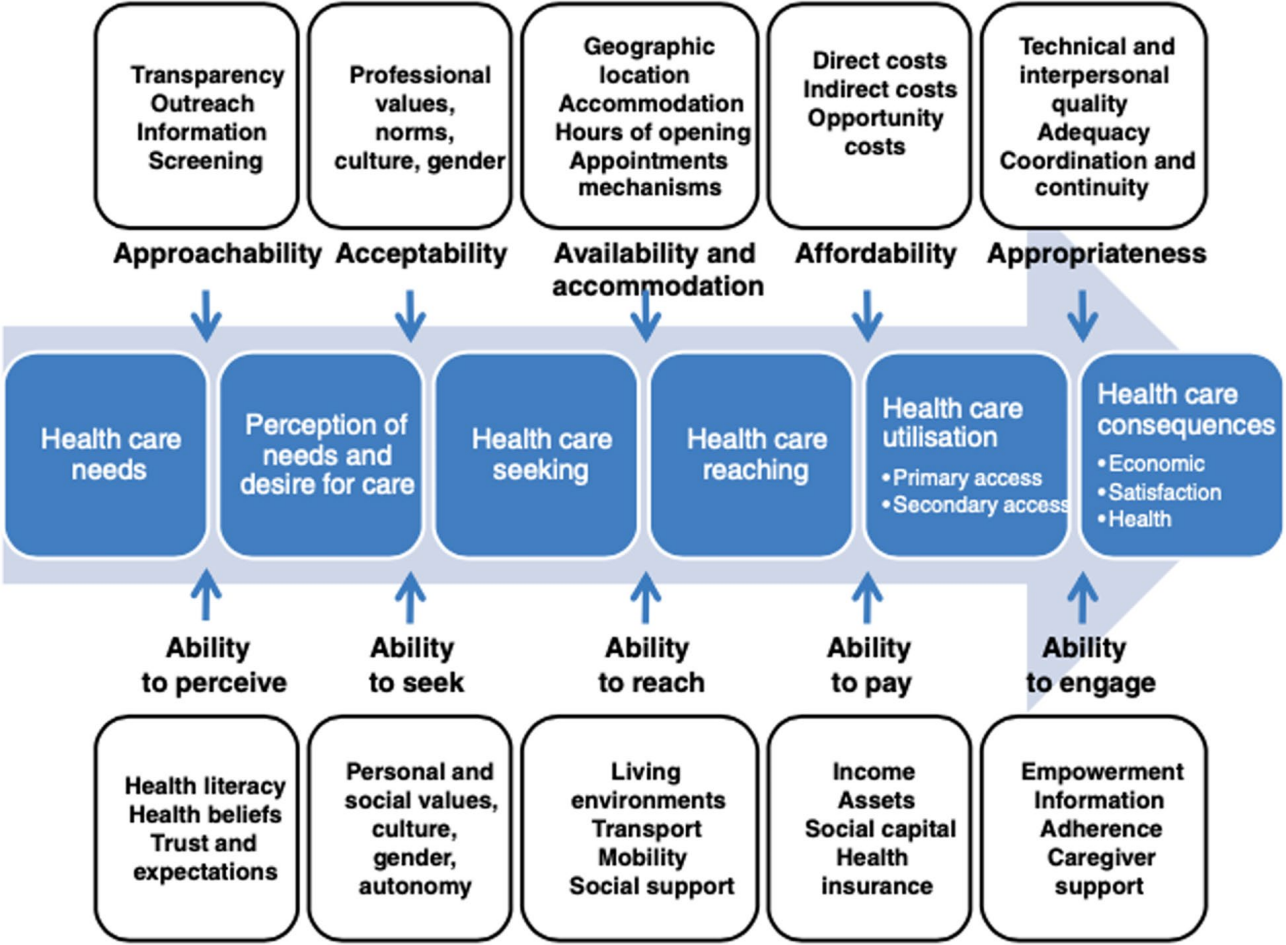
Levesque et al.'s (2013) patient-centred conceptual framework of access to healthcare

The CANARY study was informed by the Levesque patient-centred conceptual framework of access to health care (Levesque, Harris, & Russell 2013). This model has been used successfully in various healthcare settings to assess and identify barriers to access (Cu, Meister, Lefebvre, & Ridde, 2021). Levesque and colleagues defined access as the opportunity to have health care needs fulfilled and postulated that although patients have a right to health care in theory, and while services may exist, access may be restricted at any step in the process of achieving access. Healthcare access was further postulated as encompassing the possibility to choose acceptable and effective services, while the opportunity to utilize only services of poor quality was theorized as a restriction to access. Building on previous models of access to health care (Andersen 1995; Gulliford et al. 2002; McIntyre, Thiede, & Birch 2009; Penchansky & Thomas 1981; Ricketts & Goldsmith 2005), the Levesque model delineates patient and service-related factors that interact to influence the process of accessing health care, including seeking access, which culminates in healthcare consequences, including satisfaction. Most pertinent to this study, seeking access is impacted by patients’ values and autonomy, and satisfaction is impacted by the appropriateness of the services provided based on technical and interpersonal quality. See Fig. 1.

### Participants

For the purposes of our study, a sub-sample of participants from the CANARY study who indicated that they were currently authorized to use cannabis for medical purposes in Canada and had used cannabis in the past 30 days was identified (*n*= 237).

### Study measures

The CANARY study utilized an investigator-developed survey created in consultation with research partners, key policy stakeholders, and knowledge users from provincial and national patient advocacy organizations, who provided feedback on survey drafts and helped identify patients to pilot the survey. Survey items related to characteristics of cannabis products and services deemed important, and satisfaction with cannabis products and services, were drawn from previous studies and industry quality standards (Belle-Isle et al. 2014; Bottorff et al. 2011; Capler, Prosk, & Leung, 2013; Lucas 2012; Walsh et al. 2013; Ware, Ducruet, & Robinson 2006). The satisfaction-related scales were adapted from the Health Services Quality Scale (HSQS), a measure of perceived quality of health services (Dagger, Sweeney, & Johnson 2007). The HSQS has been found to be reliable and valid in samples from private outpatient oncology clinics and general practice clinics and is applicable to high-involvement, high-contact, ongoing service (Dagger et al. 2007). Specifically, participants were asked to rate, on a 5-point Likert scale, items related to the following six dimensions of products and services from different sources: overall satisfaction; quality of cannabis products; quality of care and services; expertise and support; administration and accessibility; and affordability of cannabis. Quality of cannabis products was further broken down into presentation, potency, strains, products (i.e., availability of cannabis edibles, tinctures, etc.), effectiveness, and overall product satisfaction. An additional file shows all dimensions and related items (see Additional file 1). A “not applicable” response option was provided to capture instances where the item was not relevant for individual sources.

### Statistical analyses

We conducted two sets of analyses. The first assessed source status, with respondents selecting sources of cannabis they were currently using from a list of potential sources available at the time of the study, including legal sources (i.e., Health Canada’s supplier, licensed personal production, licensed designated producer, and LPs) and illegal sources (i.e., dispensaries, personal production without a license, close friends or family, acquaintance or dealer, and unfamiliar street source). To examine differences between study participants who were using only legal products and those using illegal products, participants were categorized as currently accessing cannabis from either: (1) only legal sources (“legal-only source status”); or (2) illegal sources +/− legal sources (“any-illegal source status”). Participants that used only illegal sources (*n*=41), as well as those using both legal and illegal sources (*n*=77), were included in the “any-illegal source status” group (*n*=118).

We examined the differences between participants’ source status in relation to sociodemographic characteristics, health-related factors, and characteristics of cannabis they considered important. For the analysis, sociodemographic factors were dichotomized as shown in Table 2, and health-related factors (including health conditions and reasons for cannabis use) were dichotomized as “yes” vs. “no”. Participants were asked to select all that applied. To examine bivariate associations between the explanatory variables of interest and source status, simple logistic regressions were constructed, and 95% confidence intervals (CI) were calculated. Only complete cases were included; missing data was less than 5%. An a priori defined model-building protocol was then used, based on examination of the Akaike Information Criterion (AIC) and *p*-values, to construct an explanatory multivariate logistic regression model. A full model was constructed that included all variables with *p* <0.10 in bivariate analyses. The final multivariate model selected was the one with the lowest AIC score. We assessed multicollinearity using the Variance Inflation Factor. All *p*-values were two-sided, with *p* <0.05 being deemed significant.

In the second set of analyses, the outcomes of interest across all participants (i.e., regardless of source status) were satisfaction with various dimensions of cannabis products and services provided by the sources they indicated currently using, as well as the difference in satisfaction between legal and illegal sources. Summary statistics were conducted on the satisfaction scores given for each dimension. Composite scores were calculated as the average of scores for all items related to each dimension. Composite scores were not calculated for participants with missing data, including if they selected the “not applicable” response option. Legal and illegal sources were separated into two groups and Wilcoxon rank sum tests were used to compare satisfaction ratings for legal and illegal sources on each dimension of cannabis products and services. Analyses were conducted using 3.3.1 of R in version 0.99.903 of RStudio.

## Results

In our study sample (*n*=237), 75% were authorized under the MMAR and 25% were authorized under the MMPR. Just over 60% of participants were male, the median age was 48 years, and 85.7% described their ethnic background as White. Most of the participants were living in Ontario (38.9%) or British Columbia (28.3%). The majority reported an annual income above $20K (69.2%) and 75.5% had post-secondary education. See Table 2 for additional details.

**Table 2:** Sociodemographic characteristics stratified by source status (*n* = 237)

**Sociodemographic characteristic**	**Legal-only ** ***n* (%)** ***n* = 119**	**Any-illegal** ***n* (%) ** ***n* = 118**	**Total** ***n* (%)** ***n*=237**
*Median age*			
≥48	65 (54.6)	58 (49.2)	123 (51.9)
<48	54 (45.4)	60 (50.8)	114 (48.1)
*Sex*			
male	70 (58.8)	76 (64.4)	146 (61.6)
female	49 (41.2)	42 (35.6)	91 (38.4)
*Ethnicity*			
White	103 (86.6)	100 (84.7)	203 (85.7)
other	16 (13.4)	18 (15.3)	34 (14.3)
*Residence*			
urban/suburban	81 (68.1)	90 (76.3)	171 (72.2)
rural	38 (31.9)	28 (23.7)	66 (27.8)
*Province*			
Ontario	46 (38.7)	46 (39.0)	92 (38.8)
British Columbia	35 (29.4)	32 (27.1)	67 (28.3)
Prairies	17 (14.3)	24 (20.3)	41 (17.3)
Atlantic	17 (14.3)	8 (6.8)	25 (10.5)
Quebec	4 (3.4)	8 (6.8)	12 (5.1)
*Income*			
≥$20,000	80 (67.2)	84 (71.2)	164 (69.2)
<$20,000	39 (32.8)	34 (28.8)	73 (30.8)
Employment^*^			
employed	44 (37.0)	46 (39.0)	90 (38.0)
other	75 (63.0)	72 (61.0)	147 (62.0)
*Education*			
≥post-secondary	92 (77.3)	87 (73.7)	179 (75.5)
<post-secondary	27 (22.7)	31 (26.3)	58 (24.5)

^*^Employment includes full-time, part-time, casual, and self-employment

Pain was the most prevalent medical condition, reported by 70.5% of all study participants, followed by arthritis (43.5%) and mental health conditions (40.1%). The most common reason participants reported for using medical cannabis was pain relief (92.4%), followed by sleep issues (68.8%) and mental health (60.8%). See Table 3 for further details.

**Table 3:** Descriptive statistics of study sample’s health-related factors (*n* = 237)

**Health-related factors**	**Legal-only** ***n* (%) ** ***n* = 119**	**Any-illegal ** ***n* (%) ** ***n* = 118**	**Total** ***n* (%) ** ***n*=237**
*Medical conditions*			
Pain	85 (71.4)	82 (69.5)	167 (70.5)
Arthritis	56 (47.1)	47 (39.8)	103 (43.5)
Mental health	44 (37.0)	51 (43.2)	95 (40.1)
Respiratory	25 (21.0)	23 (19.5)	48 (20.2)
Miscellaneous	24 (20.2)	24 (20.3)	48 (20.2)
Nervous system	18 (15.1)	28 (23.7)	46 (19.4)
Gastrointestinal	19 (16.0)	22 (18.6)	41 (17.3)
Cardiovascular	18 (15.1)	16 (13.6)	34 (14.3)
Endocrine	14 (11.8)	14 (11.9)	28 (11.8)
Cancer	9 (7.6)	7 (5.9)	16 (6.8)
HIV/AIDS	5 (4.2)	6 (5.1)	11 (4.6)
*Reasons for use*			
Pain relief	109 (91.6)	110 (93.2)	219 (92.4)
Sleep	80 (67.2)	83 (70.3)	163 (68.8)
Mental health	72 (60.5)	72 (61.0)	144 (60.8)
Well-being	58 (48.7)	70 (59.3)	128 (54.0)
Inflammation*	50 (42.0)	67 (56.8)	117 (49.4)
Nausea and vomiting	58 (48.7)	58 (49.2)	116 (48.9)
Loss of appetite and weight loss	44 (37.0)	49 (41.5)	93 (39.2)
Spasms	15 (12.6)	17 (14.4)	32 (13.5)
Miscellaneous	11 (9.2)	18 (15.3)	29 (12.2)

Respondents were able to select more than one medical condition and reason for use

^*^Difference between legal only and any-illegal groups significant at *p* < 0.05

Among all participants, the most widely used cannabis source was personal production with a license (40.9%), followed by dispensaries (32.1%) and LPs (26.6%). Among legal sources accessed, personal production with a license accounted for 44.7% and LPs for 29.0%. Dispensaries comprised 46.3% of illegal sources accessed, and acquaintances or dealers accounted for 23.2%. Few participants were producing cannabis without a license or accessing from an unfamiliar street source. See Table 4 for additional details.

**Table 4:** Use of different cannabis sources by all participants (*n* = 237)

**Sources used**	**Total ** ***n* (%)**
*Legal*	
Health Canada supplier	23 (9.7)
Designated producer	34 (14.3)
Personal production (with license)	97 (40.9)
Licensed producer (LP)	63 (26.6)
*Illegal*	
Medical cannabis dispensary	76 (32.1)
Personal production (without license)	11 (4.6)
Close friend/family	29 (12.2)
Acquaintance/dealer	38 (16.0)
Unfamiliar street source	10 (4.2)

Respondents were able to select more than one source

### Source status

The sample was equally divided between legal-only source status (*n*=119) and any-illegal source status (*n*=118). There were no statistically significant differences between participants categorized in the legal-only and any-illegal source status groups with regard to sociodemographic characteristics and medical conditions. In terms of reasons for use, the legal-only source status group was significantly less likely to report inflammation in both the bivariate (OR = 0.55; 95% CI = 0.33–0.92) and multivariate regression analysis (adjusted odds ratio=0.53; 95% CI = 0.31–0.88) (*p* <0.05 for both bivariate and multivariate regression). There were no significant differences between source status groups for any other reasons for use.

There were several significant differences between the legal-only and any-illegal source status groups regarding the degree of importance assigned to specific characteristics of cannabis products and services. Participants in the any-illegal cannabis source status group were significantly more likely to consider the following features important: available at a dispensary (OR: 4.55; 95% CI: 2.56–8.33), ability to select strain and dosage (OR: 4.17; 95% CI: 2.13–8.33), ability to observe and smell cannabis (OR: 2.72; 95% CI: 1.30–3.85), pesticide-free product (OR: 2.27; 95% CI: 1.09–4.55), available in small quantities (OR: 2.17; 95% CI: 1.28–3.57), and access to a variety of strains (OR: 2.04; 95% CI: 1.05–4.00) (see Table 5).

**Table 5:** Descriptive statistics and association between source status and characteristics deemed important (*n* = 237)

**Characteristic**	**Legal-only ** ***n* (%) ** ***n* = 119**	**Any-Illegal** ***n* (%) ** ***n* = 118**	**Unadjusted OR** **(95%CI)**
Access to preferred strains	105 (88.2)	103 (87.3)	0.92 (0.42–2.00)
Access to a variety of products	100 (84.0)	99 (83.9)	0.99 (0.49–2.00)
Organically grown	96 (80.7)	88 (74.6)	0.70 (0.19–1.30)
Free of pesticides	93 (78.2)	105 (89.0)	2.27 (1.09–4.55)^*^
Access to a variety of strains	90 (75.6)	102 (86.4)	2.04 (1.05–4.00)^*^
Provided in trimmed form	88 (73.9)	92 (78.0)	1.25 (0.68–2.27)
Free of microbial contaminants	85 (71.4)	94 (79.6)	1.56 (0.86–2.86)
Able to select strain and dosage	76 (63.9)	104 (88.1)	4.17 (2.13–8.33)^**^
Able to observe and smell	66 (55.5)	87 (73.7)	2.72 (1.30–3.85)^**^
Standardized levels of ingredients	61 (51.3)	72 (61.0)	1.49 (0.88–2.50)
Available in large quantities	59 (49.6)	72 (61.0)	1.59 (0.95–2.63)
Sent to home	57 (47.9)	61 (51.7)	1.16 (0.70–1.92)
Available in a dispensary	52 (43.7)	92 (78.0)	4.55 (2.56–8.33)^**^
Available in small quantities	51 (42.9)	73 (61.9)	2.17 (1.28–3.57)^**^
Available in a pharmacy	38 (31.9)	49 (41.5)	1.52 (0.89–2.56)
Provided in milled form	10 (8.4)	5 (4.2)	0.48 (0.15–1.44)
Other	9 (7.6)	12 (10.2)	0.39 (0.56–3.45)

Bivariate logistic regression analyses

*OR*, odds ratio; *CI*, confidence interval

^*^p <0.05

^**^p <0.01

### Satisfaction with legal and illegal sources

Participants indicated their satisfaction with medical cannabis products and services for each source they were currently using (irrespective of “source status”). Significant differences between legal and illegal sources were found for all service-related dimensions. Illegal sources were rated significantly higher than legal sources for quality of care and service (*p*=0.004), expertise and support (*p*=0.025), and administration and accessibility (*p*=0.008) (see Table 6).

**Table 6:** Comparison of ratings of legal and illegal sources on service-related dimensions of care (*n* = 237)

**Dimension**	Median of mean scores^*^ (IQR)		
	**Legal sources**	**Illegal sources**	***p*-value**
Quality of care	3.67 (2.60–4.48)	4.02 (3.40–4.60)	0.004
Expertise and support	3.25 (2.43–4.18)	3.64 (3.13–4.34)	0.025
Administration and accessibility	3.67 (2.80–4.50)	4.00 (3.43–4.68)	0.008

Wilcoxon rank-sum test

*IQR*, interquartile range

^*^Mean score on scale of 1 to 5 (1 = very poor to 5 = very good)

Close friend/family, designated producer, and dispensaries were given the highest mean scores for quality of care (4.33, 4.25, and 4.09, respectively) and expertise and support (3.66, 4.12, and 4.01, respectively). Designated producers, dispensaries, and production without a license were given the highest scores for administration and accessibility (4.22, 4.13, and 4.58, respectively) (see Table 7).

**Table 7:** Service-related satisfaction means scores for sources currently using (*n*= 237)

	**Quality of care**			**Expertise and support**			**Administration and accessibility**			
*Sources:*	n	Mean	(SD)	n	Mean	(SD)	n	Mean	(SD)	
*Legal sources*										
Health Canada supplier	23	2.08	(0.97)	23	2.21	(0.98)	22	3.09	(0.90)	
Designated producer	33	4.25	(1.17)	34	4.12	(1.00)	34	4.22	(1.06)	
Personal production (with license)	n/a	n/a	n/a	n/a	n/a	n/a	89	3.56	(1.28)	
Licensed producer	61	3.41	(1.00)	63	3.18	(1.01)	63	3.34	(0.95)	
*Illegal sources*										
Medical cannabis dispensary	76	4.09	(0.90)	76	4.01	(0.80)	76	4.13	(0.78)	
Personal production (no license)	n/a	n/a	n/a	n/a	n/a	n/a	9	4.58	(0.49)	
Close friend/family	29	4.33	(0.68)	29	3.66	(0.88)	28	3.95	(0.87)	
Acquaintance/dealer	38	3.35	(1.07)	37	2.85	(1.06)	38	3.69	(1.10)	
Unfamiliar street source	7	2.12	(0.85)	8	2.27	(0.94)	9	2.51	(1.19)	

^a^Mean score on scale of 1 to 5 (1 = very poor to 5 = very good)

*n/a*, not applicable. Questions for quality of care and expertise and support pertain to service provided by others; therefore, participants were not asked these questions regarding personal production with license and personal production with no license

No significant differences were found between legal and illegal sources for overall satisfaction, affordability, or product satisfaction (see Tables 8 and 9 for further details). Cronbach’s alpha scores for the adapted satisfaction-related scales used in this study were found to be highly reliable in the study sample, ranging from 0.82 to 0.87.

**Table 8:** Product satisfaction mean scores for sources currently using (*n*= 237)^a^

Product Satisfaction						
*Sources:*	Overall	Presentation	Potency	Strains	Products	Effectiveness
*Legal sources*	Mean (SD)	Mean (SD)	Mean (SD)	Mean (SD)	Mean (SD)	Mean (SD)
Health Canada supplier (*n* = 23)	2.61 (1.37)	2.94 (1.20)	2.61 (1.20)	1.39 (0.90)	1.35 (1.03)	2.87 (1.29)
Designated producer (*n* = 34)	4.62 (0.82)	4.52 (0.64)	4.68 (0.59)	4.13 (0.96)	2.89 (1.67)	4.77 (0.55)
Personal production (with license) (*n* = 97)	4.57 (0.93)	4.42 (0.91)	4.31 (1.09)	4.07 (1.29)	4.08 (1.37)	4.43 (1.05)
Licensed producer (*n* = 63)	3.70 (1.13)	3.87 (0.85)	3.62 (1.05)	2.74 (1.15)	1.49 (1.05)	3.62 (1.17)
*Illegal sources*						
Medical cannabis dispensary (*n* = 76)	4.28 (0.99)	4.29 (0.67)	4.33 (0.77)	4.17 (0.93)	4.01 (1.26)	4.49 (0.84)
Personal production (no license) (*n* = 11)	4.55 (0.69)	4.52 (0.55)	4.55 (0.69)	4.27 (1.42)	4.64 (0.92)	4.91 (0.30)
Close friend/family (*n* = 29)	4.24 (0.79)	4.08 (0.73)	4.00 (0.93)	3.07 (1.42)	2.69 (1.73)	4.21 (0.94)
Acquaintance/dealer (*n* = 38)	3.63 (0.91)	3.58 (0.86)	3.68 (1.07)	2.65 (1.11)	1.68 (1.07)	3.97 (1.15)
Unfamiliar street source (*n* = 10)	3.30 (1.06)	2.83 (0.98)	3.20 (1.23)	2.15 (1.25)	1.50 (0.85)	3.40 (1.27)

^a^Mean score on scale of 1 to 5 (1 = very poor to 5 = very good)

**Table 9:** Overall satisfaction and affordability mean scores for sources currently using (*n*= 237)

**Overall satisfaction**	**Mean**^a^	**(SD)**	**Affordability**	**Mean**^b^	**(SD)**
*Legal sources*			*Legal sources*		
Health Canada supplier (*n* = 23)	2.48	(1.28)	Health Canada supplier (*n* = 21)	2.62	(1.16)
Designated producer (*n* = 34)	4.65	(0.73)	Designated producer (*n* = 32)	4.06	(1.39)
Personal production (with license) (*n* = 97)	4.54	(1.02)	Personal production (with license) (*n* = 84)	3.66	(1.71)
Licensed producer (*n* = 63)	3.21	(1.43)	Licensed producer (*n* = 63)	2.06	(1.34)
*Illegal sources*			*Illegal sources*		
Medical cannabis dispensary (*n* = 76)	4.22	(0.96)	Medical cannabis dispensary (*n* = 75)	2.89	(1.49)
Personal production (no license) (*n* = 11)	4.46	(0.82)	Personal production (no license) (*n* = 8)	5.00	(0.00)
Close friend/family (*n* = 29)	4.28	(0.75)	Close friend/family (*n* = 26)	3.35	(1.55)
Acquaintance/dealer (*n* = 38)	3.61	(1.08)	Acquaintance/dealer (*n* = 38)	2.47	(1.29)
Unfamiliar street source (*n* = 10)	2.60	(1.35)	Unfamiliar street source (*n* = 9)	1.22	(0.44)

^a^Mean score on scale 1 to 5 (1 = completely unsatisfied and 5 = completely satisfied)

^b^Mean score on a scale of 1 to 5 (1 = strongly disagree; 5 = strongly agree that the cannabis and associated costs are affordable)

## Discussion

This study occurred during a unique time of overlapping regulations for medical cannabis, providing valuable insights into authorized patients’ experiences during a transition between regulatory frameworks. The study findings provide an historical perspective regarding medical cannabis in Canada, filling a knowledge gap regarding the transitional period between the MMAR and MMPR, and the initiation of the LP system in Canada. These findings hold relevance today, given the continued use of both legal and illegal sources by individuals using cannabis for medical purposes. Further, the findings provide valuable insights into the characteristics of cannabis products and services that may lead individuals to use legal sources and dissuade the use of illegal sources—dynamics of concern for jurisdictions now legalizing medical or recreational cannabis use or both.

A major finding of our study was that while all participants had the authorization to access legal sources of medical cannabis, 50% accessed cannabis from illegal sources. This represents a substantial reduction compared to previous research that found 80% of individuals with authorization under the MMAR sought medical cannabis from illegal sources (Belle-Isle et al. 2014); however, it still represents a sizable proportion of authorized medical cannabis users. This corresponds to findings from an Australia study where 64.6% of cannabis users with a prescription for medical use accessed cannabis from both legal and illegal sources (Lintzeris et al. 2022). Similar to previous research in Canada (Walsh et al. 2013), we found no significant differences between participants who used only legal sources and those who use illegal sources in terms of sociodemographic characteristics, medical conditions, or reasons for use. These findings suggest that the use of legal and illegal sources is not indicative of differing healthcare needs; rather, there are other patient- and health service-related factors that may account for the use of legal and illegal sources.

Notably, this was the first study to apply the Levesque model of healthcare access (Levesque et al. 2013) as a theoretical lens to explore access to medical cannabis. In privileging patients’ access experiences, the Levesque model contributes to our understanding of factors that may impact use of illegal and legal sources, and what constitutes reasonable access from the perspective of patients. In terms of patient-related factors, according to the Levesque model personal values and autonomy impact individuals’ decisions during the seeking stage of health care access. In our study, individuals using legal and illegal sources held significantly different values related to specific characteristics of cannabis products and services that may have accounted for their use of those sources. In terms of service-related factors, the Levesque model conceives satisfaction with healthcare services as a consequence of the access experience. While the Levesque model is linear, we suggest that satisfaction with a healthcare access experience may also influence the decision to use that source in the future. In our study, participants reported significantly higher levels of satisfaction for illegal sources than for legal sources on all service dimensions (i.e., quality of care and service, expertise and support, and administration and accessibility). Based on the findings of this study, it is possible that the services provided by illegal sources may, in part, have accounted for the use of these sources.

Regarding patient-related factors, our study found that compared to individuals who accessed medical cannabis from only legal sources, individuals accessing from illegal sources placed significantly greater value on having access to a variety of strains, being able to directly observe and smell cannabis products, as well as having the autonomy to select the strain and dosage. The importance of having access to specific strains has been identified in previous research (Capler et al. 2017, Lankenau et al, 2018), with type of strain being reported to be an important determinant of perceived effectiveness by medical cannabis consumers (Sexton, Cuttler, Finnell, & Mischley 2016; Walsh et al. 2013). Further, there is emerging evidence of a subjective and theoretical differential therapeutic action of distinct cannabis strains across various symptoms and health conditions (Cuttler et al. 2018; Russo 2011; Sawler et al. 2015). Some research also suggests that visual and olfactory inspection can provide valuable information about the potential effects of a particular strain. For example, terpenes, which provide the characteristic smell of cannabis and vary by type and amount across different strains, may be partially responsible for different physiological and cognitive effects (Blasco-Benito et al. 2018; LaVigne et al., 2021, Lewis et al. 2018; Russo & Marcu 2017; Sawler et al. 2015). Furthermore, the ability to inspect and select strains and dosages reflects a higher level of autonomy, and previous research has found that individuals who accessed illegal sources of medical cannabis value autonomy in their healthcare decisions (Bottorff et al. 2011; Fainzang 2013; Hanna & Hughes 2011). Study participants accessing from illegal sources also placed greater importance on cannabis that was available through a dispensary, available in small quantities, and pesticide-free. Concerns about possible toxic effects from pesticide use in the production of cannabis have also been validated by studies that have found high levels of pesticide residue can be transferred into the smoke of cannabis when it is combusted (Dryburgh et al. 2018; Raber, Elzinga, & Kaplan 2015; Sullivan, Sytze, & Raber, 2013; Taylor & Birkett 2020). Although concerns have been raised about pesticide use on cannabis obtained from both LPs and dispensaries (Brown 2017; Eykelbosh 2021; Robertson & McArthur 2016), individuals using medical cannabis in Canada have reported trusting the quality of cannabis acquired from dispensaries (Bottorff et al. 2011; Capler et al. 2017). Despite increases in use of legal sources by non-medical cannabis users in Canada since legalization (Wadsworth et al, 2023), only 29.3% perceived legal cannabis to be higher quality than illegal cannabis (Wadsworth et al, 2022). In a qualitative study of non-medical cannabis consumers in Newfoundland and Labrador, participants reported that they considered both legal and illegal sources to be safe and that there were trustworthy illegal sources offering high-quality, safe products. Further, individuals who had experience using illegal sources indicated that the products were of superior quality compared to licensed legal sources (Donnan et al., 2022).

Findings of this study suggest that for some patients, the products and services found in illegal sources were better able to meet their needs related to medical cannabis. At the time of our study, many of the characteristics of cannabis products and services that were deemed significantly more important by participants using illegal sources were offered almost exclusively by illegal sources. For example, when LPs were first introduced, they had a limited selection of strains and products available compared to illegal sources. Illegal sources also allowed individuals to directly inspect products, whereas individuals accessing cannabis through mail-order from LPs were reliant on images and descriptions provided online. Within the legal medical cannabis program, healthcare providers determine the allowable daily dosage; in contrast, individuals accessing medical cannabis from illegal sources had a greater level of autonomy to determine the amount of cannabis they purchased, produced, and consumed. Additionally, whereas LPs stipulate a minimum amount of cannabis that can be ordered (i.e., 15 grams), many illegal sources sell cannabis in smaller amounts, allowing access for patients with limited funds. Furthermore, at the time of our study, illegal sources of medical cannabis offered a highly personalized and in-person experience in contrast to legal sources, which only provided online sales and support. For example, friends and family, which received the highest scores for quality of care and service, also provide the most personalized experience of cannabis access. Dispensaries, which were the most utilized illegal source of medical cannabis in our study and were found to be widely accessed by individuals using medical cannabis in national studies prior to cannabis legalization (Government of Canada 2017, 2018a; Walsh et al. 2013), were designed to cater to the needs of individuals using cannabis for medical purposes. Dispensaries were highly rated on service dimensions in our study. A previous Canadian study similarly reported that dispensaries were equally or more favorably evaluated on service-related dimensions, including safety, efficiency, and feeling respected, compared to other legal and illegal sources (Capler et al. 2017).

Since this study was conducted, there have been some substantial changes to the types and uses of legal and illegal sources of medical cannabis. While the types of legal sources of medical cannabis have remained the same, the number of LPs and the variety of strains and products they offer have grown substantially (Government of Canada 2019a, 2022b). Despite those changes, active client registration with LPs has steadily dropped since 2018, from 345,520 to 264,686 in 2021 (Government of Canada 2022c); however, the proportion of medical users reporting the use of LPs has remain stable at approximately 20% since 2017 (Government of Canada 2021a). At the same time, licenses for personal and designated production have increased steadily (Government of Canada 2022c), which may partly reflect the lack of affordable access offered by LPs ( Medical Cannabis Canada 2020). With respect to illegal sources, there has been a dramatic decrease in their use by individuals self-reporting use of cannabis for medical purposes. For example, access from dispensaries fell steeply from 28% in 2018 to 6% in 2020 (Government of Canada 2021b), coinciding with the closure of most dispensaries during this time (Mahamad, Wadsworth, Rynard, Goodman, & Hammond 2020). Access from friends also decreased from 36% in 2018 to 11% in 2021.

The new sources of legal non-medical cannabis introduced with cannabis legalization (i.e., retail storefronts, online sales, and personal production of cannabis) have considerably impacted where individuals can legally access cannabis, regardless of their intention to use it for medical or non-medical purposes. It appears that access from non-medical legal sources has replaced access from illegal sources of medical cannabis to a large extent. Legal non-medical storefronts were accessed by 53% of individuals reporting medical use of cannabis in 2021, up from 44% in 2020 (Government of Canada 2021a; Medical Cannabis Canada 2020). Considering our findings, individuals using cannabis for medical purposes may be drawn to legal non-medical storefronts due to the ability to inspect and select the products and the in-person service available in such settings. Use of legal non-medical online sources by individuals using cannabis for medical purposes has also been sharply increasing, with 38% reporting use of this source in 2021, up from 23% in 2020 (Government of Canada 2021a), suggesting medical cannabis users are taking advantage of all the legal sources available to them, regardless of whether they cater to medical use. Legal non-medical cannabis sources are not permitted to explicitly offer products and services that address medical use, including providing information or advice about strain selection for symptom management, which may impact the quality of service for medical cannabis users. In Colorado, where there are both legal medical and recreational retail storefronts, medical cannabis patients consider information provided by medically focused storefronts to be of higher quality than information provided by recreational storefronts (Alon et al. 2021). A national cross-sectional study of legal cannabis retail storefronts in the USA found that individuals working at retail storefronts in states with more medicalized programs vs. less medicalized programs were significantly more likely to provide recommendations to customers using cannabis for medical purposes based on training provided by employers and physician input (Merlin et al. 2021). Legal medical storefronts are still not part of Canada’s medical cannabis system, creating a potential void in medical cannabis access.

As regulations for medical cannabis continue to evolve in Canada and internationally, the findings from this study can inform future policy and research, with the goal of providing reasonable access to cannabis for medical purposes. This study underscores the importance ensuring individuals who use cannabis for medical purposes have access to legal sources of cannabis that incorporate the characteristics they value, respect their autonomy, and are appropriate for their unique needs. Although individuals using cannabis for medical purposes have increased their use of both medical and non-medical legal cannabis sources, it is unclear whether these options are currently meeting their needs. Questions have been raised about retaining a separate medical cannabis program under the Cannabis Act (Geary 2018; Task Force on Cannabis Legalization and Regulation, 2016). To inform the upcoming legislative review of the Cannabis Act and the medical cannabis program, future research should identify the characteristics of products and services that are important to individuals who use cannabis for medical purposes and assess whether they are present in the legal medical program. It would also be instructive to evaluate patient satisfaction with the products and services provided by legal medical cannabis sources to determine whether these sources provide reasonable access. Currently, legal medical cannabis sources that are meeting patients’ needs, including personal and designated production, should be retained, and other sources that are not currently legal, such as medical cannabis storefronts, should be added to the medical cannabis program.

### Limitations

While this study offers important insights into access to medical cannabis in Canada, there are limitations related to the online and cross-sectional design, as well as sampling and recruitment techniques. As a result of the online survey administration and recruitment through specific disease and medical cannabis networks, the sample may not be representative of the Canadian population legally accessing medical cannabis. Response and selection biases may have also influenced the results, resulting in either overly positive or negative responses to questions related to satisfaction with cannabis products and services from legal and illegal sources. Our analysis did not compare individuals using only illegal sources with those using only legal sources and those using both legal and illegal sources as that was not the focus of this study. With regard to instrumentation, it is possible that there were other factors associated with the use of legal and illegal sources that we did not assess. We did not measure whether participants had experience with the characteristics of cannabis products and services they deemed important and how such experience, or lack thereof, may have impacted their responses. Lastly, we did not directly assess whether the legality of cannabis sources was an important consideration for those using only legal or any illegal sources; such analyses may provide relevant information in future research. A comparison of individual legal and illegal sources would also be instructive.

## Conclusion

As the 5-year review of Canada’s medical cannabis program under the Cannabis Act commences, our study findings highlight some of the key characteristics of cannabis products and services that are valued by patients and should be incorporated into the legal medical cannabis program to promote the use of legal medical sources and deter the use of illegal sources. Our findings also shed light on what comprises reasonable access to medical cannabis from the perspective of patients, including having more choice, autonomy, and in-person support throughout the access process with regard to products and services. While the findings of this study pertain specifically to the medical use of cannabis in Canada, they may also be instructive for understanding the use of illegal cannabis sources for non-medical use in Canada and provide insight for other jurisdictions implementing cannabis regulations for both medical and non-medical use.

## Supplementary Information

**Additional file 1.** 

## Data Availability

The datasets used and/or analyzed during the current study are available from the corresponding author on reasonable request.

## Abbreviations

AIC: Akaike Information Criterion
ACMPR: Access to Cannabis for Medical Purposes Regulations
CANARY: Cannabis Access Regulations Study
CI: Confidence interval
LP: Licensed producer
MMAR: Marihuana Medical Access Regulation
MMPR: Marihuana for Medical Purposes Regulations
OR: Odds ratio
