# Supplementary material for: Reasonable access: important characteristics and perceived quality of legal and illegal sources of cannabis for medical purposes in Canada
Source: J Cannabis Res. 2023 Jun 9;5:18. doi: 10.1186/s42238-023-00185-w (PMC10251568; doi:10.1186/s42238-023-00185-w)
Supplement: Supplementary file 1 — Additional file 1. [file 42238_2023_185_MOESM1_ESM.pdf]

Additional File 1. Perceived quality of cannabis products and services: dimensions and related items

**1. Quality of medical cannabis products** (1 = very poor to 5 = very good)
• Presentation (humidity; appearance; grind)<br>• Potency (THC content)<br>• Strains (selection of strains; availability of preferred strains)<br>• Availability of cannabis products (edibles, tinctures, etc.)<br>• Effectiveness<br>• Overall product satisfaction
2. **Quality of care and service** (1 = strongly disagree to 5 = strongly agree)
• They always listen to what I have to say;<br>• They understand my needs;<br>• There are opportunities for me to provide feedback;<br>• I find it easy to talk with them; they answer my questions;<br>• They explain things in a way that I can understand;<br>• I believe they care about me;<br>• I talk with them about things that are happening in my life, not just about my medical condition or cannabis products;<br>• I get personalized attention from them; and<br>• I have built a good relationship with them.
**3. Quality of expertise and support** (1 = strongly disagree to 5 = strongly agree)
• I can rely on them to be well trained and qualified;<br>• They carry out their tasks competently;<br>• They provide thorough explanations about the medicine and different ways to take it;<br>• They make good recommendations for strains and products most appropriate for my particular condition and symptoms;<br>• They help me to keep track of the cannabis strains and products I have used; and<br>• They support me to meet my needs around medical cannabis.
**4. Administrative quality and accessibility** (1 = strongly disagree to 5 = strongly agree)
• I receive medical cannabis in a timely manner;<br>• Generally, appointments run on time;<br>• They have responded to my calls or other inquiries in a timely manner;<br>• The registration procedures are accurate;<br>• I believe is well managed;<br>• The hours of operation meet my needs;<br>• The location is convenient;<br>• The options for accessing medical cannabis meet my needs (i.e., mail, in-person); and<br>• I feel safe getting cannabis through this source
**5. Affordability** (1 = strongly disagree; 5 = strongly agree)
• The cannabis and associated costs are affordable
**6. Overall Satisfaction (**1 = completely unsatisfied and 5 = completely satisfied)
• How would you rate your overall satisfaction with the following sources
